# Protease-Activated Receptor 4 Induces Bladder Pain through High Mobility Group Box-1

**DOI:** 10.1371/journal.pone.0152055

**Published:** 2016-03-24

**Authors:** Dimitrios E. Kouzoukas, Fei Ma, Katherine L. Meyer-Siegler, Karin N. Westlund, David E. Hunt, Pedro L. Vera

**Affiliations:** 1 Research and Development, Lexington Veterans Affairs Medical Center, Lexington, Kentucky, United States of America; 2 Department of Natural Sciences, St. Petersburg College, St. Petersburg, Florida, United States of America; 3 Department of Physiology, University of Kentucky, Lexington, Kentucky, United States of America; 4 Department of Surgery, University of Kentucky, Lexington, Kentucky, United States of America; 5 Saha Cardiovascular Research Center, University of Kentucky, Lexington, Kentucky, United States of America; National Cancer Institute at Frederick, UNITED STATES

## Abstract

Pain is the significant presenting symptom in Interstitial Cystitis/Painful Bladder Syndrome (IC/PBS). Activation of urothelial protease activated receptor 4 (PAR4) causes pain through release of urothelial macrophage migration inhibitory factor (MIF). High Mobility Group Box-1 (HMGB1), a chromatin-binding protein, mediates bladder pain (but not inflammation) in an experimental model (cyclophosphamide) of cystitis. To determine if PAR4-induced bladder hypersensitivity depends on HMGB1 downstream, we tested whether: 1) bladder PAR4 stimulation affected urothelial HMGB1 release; 2) blocking MIF inhibited urothelial HMGB1 release; and 3) blocking HMGB1 prevented PAR4-induced bladder hypersensitivity. HMGB1 release was examined in immortalized human urothelial cultures (UROtsa) exposed to PAR4-activating peptide (PAR4-AP; 100 μM; 2 hours) or scrambled control peptide. Female C57BL/6 mice, pretreated with a HMGB1 inhibitor (glycyrrhizin: 50 mg/kg; ip) or vehicle, received intravesical PAR4-AP or a control peptide (100 μM; 1 hour) to determine 1) HMGB1 levels at 1 hour in the intravesical fluid (released HMGB1) and urothelium, and 2) abdominal hypersensitivity to von Frey filament stimulation 24 hours later. We also tested mice pretreated with a MIF blocker (ISO-1: 20 mg/kg; ip) to determine whether MIF mediated PAR4-induced urothelial HMGB1 release. PAR4-AP triggered HMGB1 release from human (*in vitro)* and mice (*in vivo*) urothelial cells. Intravesical PAR4 activation elicited abdominal hypersensitivity in mice that was prevented by blocking HMGB1. MIF inhibition prevented PAR4-mediated HMGB1 release from mouse urothelium. Urothelial MIF and HGMB1 represent novel targets for therapeutic intervention in bladder pain conditions.

## Introduction

Bladder pain commonly occurs without obvious bladder pathology and is a cardinal symptom of interstitial cystitis / painful bladder syndrome (IC/PBS), a chronic condition with unknown etiology affecting 2.7–6.5% of women in the U.S. [1]. Rodent models of bladder pain historically relied on producing pain secondary to bladder injury and inflammation [2,3]. However recent reports [4–7] show that bladder pain can be independent from bladder inflammation including one where sequestration of high-mobility group box 1 protein (HMGB1, a nuclear chromatin-binding protein) prevented bladder pain in a cyclophosphamide model of cystitis without affecting inflammatory indicators [6].

HMGB1 is translocated to the cytoplasm and secreted by active and passive processes during pathogenic infection or tissue injury [8]. Extracellular release of HMGB1 mediates both inflammation, by acting as a proinflammatory cytokine [9], and pain, by directly affecting neuronal activity [10]. Elevated levels are observed in inflammatory pain conditions like rheumatoid arthritis [11] and pancreatitis [12]. Blocking HMGB1 with anti-HMGB1 monoclonal antibodies ameliorates pain behaviors in rodent models of neuropathic and bone cancer pain [13,14] while injection of recombinant HMGB1 elicits pain behaviors in rodents [10]. Together these findings indicate HMGB1 plays a key role in mediating pain at multiple sites, including peripheral (e.g. organ) and central (e.g. spinal cord) levels [10].

Urothelial cells express protease-activated receptors (PARs) that are activated when proteases cleave the tethered ligand [15,16]. Interestingly, IC/PBS patients have elevated urine protease levels [17,18], which presumably may lead to greater bladder PAR activation. We recently showed that activation of urothelial PAR4 receptors triggered pain without causing overt inflammation through a macrophage migration inhibitory factor (MIF)-mediated mechanism [7]. Urothelial MIF is constitutively expressed and stored for release upon noxious stimuli to further mediate downstream inflammatory changes and pain in the bladder [19]. Since HMGB1 release can initiate pain independent of inflammation, we tested the hypothesis that HMGB1 also mediates pain in our PAR bladder pain model.

To this end, we examined PAR4-induced HMGB1 release in human (SV40-transformed) urothelial cells (UROtsa). In addition, in female mice receiving intravesical instillation of a PAR4-activating peptide (AP), we tested (1) urothelial HMGB1 release; (2) HMGB1 inhibitor antagonism of PAR4-induced bladder hypersensitivity; and (3) MIF inhibitor antagonism of PAR4-induced HMGB1 release. Our findings revealed that bladder PAR4 receptor activation elicits HMGB1 release from the urothelia through a MIF-mediated mechanism to cause bladder pain.

## Materials and Methods

### *In vitro* experiments

UROtsa cells (derived from the urothelium lining of benign human ureter immortalized with SV40; a gift of Scott H Garrett [20]) were used as an *in vitro* model of normal urothelium. Cells were plated in 24-well plates (five replicates per treatment group) at a density of 6 x 10^4^ cells/ml overnight in DMEM with 10% FBS. Cells were synchronized 1 hour in fresh DMEM (with 0.1% BSA) before replacing media with DMEM (with 0.1% BSA) containing a human PAR4-activating peptide (AYPGKF-NH2) or a scrambled control peptide (YAPGKF-NH2) at 100 μM (Peptides International, Inc., Louisville, KY). Culture medium was collected at 2 hours, and assayed for HMGB1 by western blotting.

### *In vivo* experiments

All animal experiments were approved by Lexington Veterans Affairs Medical Center Institutional Animal Care and Use Committee (VER-11-016-HAF) and performed according to the guidelines of the National Institutes of Health.

### Abdominal mechanical hypersensitivity testing

**Fig 1:**
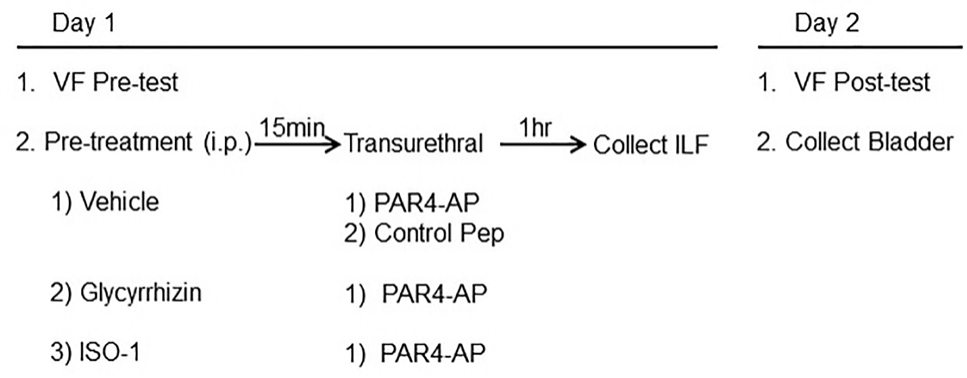
The flow chart demonstrates *in vivo* study in mice with treatments.

Abdominal mechanical hypersensitivity was tested in mice (13–17 week-old female C57BL/6; Jackson Laboratory, Bar Harbor, ME) as previously described [7]. Briefly, von Frey filaments of ascending bending force (0.008, 0.020 0.040, 0.070 g) were pressed to the lower abdominal region in trials of 10 before (baseline) and 24 hours after PAR4 peptide instillation to detect referred bladder pain. Positive response was defined as any one of three behaviors: 1) licking the abdomen, 2) flinching/jumping, or 3) abdomen withdrawal. Mice responding more than 30% to the weakest filament (0.008 g) during baseline testing were excluded from the study. The experimental design is illustrated in Fig 1.

### Intravesical instillation of PAR4 peptides and bladder collection

Isoflurane-anesthetized mice were transurethrally catheterized (PE10, 11 mm length) and drained of urine [7]. Fifteen minutes before instillation, mice received either HMGB1 antagonist [21], glycyrrhizin (50 mg/kg, ip; Calbiochem, Billerica, MA), glycyrrhizin vehicle control (10 μM NH_4_OH in sterile PBS, pH 7.4; ip), or MIF antagonist, (S,R)3-(4-hy-droxyphenyl)-4,5-dihydro-5-isoxazole acetic acid methyl ester [22] (ISO-1; 20 mg/kg, ip; EMD Bioscience, San Diego, CA; catalog 475837). Bladders were instilled with either PAR4-activating peptide (AYPGKF-NH2; 100 μM in PBS; pH 7.4, 150 μl) or a scrambled control peptide (YAPGKF-NH2; 100 μM in PBS; pH 7.4, 150 μl) and retained for 1 hour. Intravesical fluid was collected from the catheter tip, treated with protease inhibitors (Halt III; Thermo Sci., Rockford, IL), and stored at -80°C until analysis.

Twenty-four hours after instillation, mice were tested for abdominal mechanical allodynia (as above) and then anesthetized (isofluorane anesthesia). Bladders were removed, fixed in 10% formalin, and embedded in paraffin for histology (see below).

### Western blotting

Culture medium from human urothelial cells (UROtsa) was diluted 1:1 with 2X Laemmli sample buffer with 10% beta-mercaptoethanol and proteins separated using a 4–15% Mini-PROTEAN TGX precast polyacrylamide gel (Bio-Rad, Hercules, CA). We also measured HMGB1 levels in mouse intravesical fluid collected after intravesical instillation of PAR4-AP or control peptide. Intravesical fluid, using equal volume across all samples, was heated to 99°C for 20 minutes in Laemmli buffer (Bio-Rad, Hercules, CA) in non-reducing, denaturing, conditions before loading in 12% gels (Mini-PROTEAN TGX; Bio-Rad, Hercules, CA). After electrophoresis, separated proteins were transferred to a polyvinylidene difluoride membrane. For culture media, the blot was incubated with a secondary antibody (donkey anti-rabbit IR Dye 800 CW; LI-COR, Lincoln, NE) at a concentration of 1:10,000 for 1 hour. The blot was washed three times for 10 minutes and then imaged using Odyssey Imager (LI-COR). In order to test the specificity of the antibody, antibody-blocking peptide (ab18650; Abcam, Cambridge, MA) was made up in block buffer by mixing 10 μg of antibody with five times excess blocking peptide (50 μg) overnight at 4°C. The primary antibody block solution was added to the blocked blot overnight at 4°C. The secondary antibody and the imaging conditions were the same as for the Western blot. In order to document equal protein loading in all lanes, a duplicate gel was not transferred but just stained using Coomassie. In addition, the blot was stained right after transfer with the Novex® Reversible Protein Stain (IB7710; Thermo Sci., Rockford, IL), photographed, destained and blocked overnight.

HMGB1 protein bands in the intravesical fluid were visualized using a rabbit polyclonal primary antibody (ab18256; Abcam, Cambridge, MA; 1:4000 or 1 mg/ml for culture media), a biotinylated anti-rabbit secondary antibody (Vector Labs, Burlingame, CA; 1:400), streptavidin-HRP conjugates and chemiluminescent substrate (Pierce, Rockford, IL). Band densitometry was performed using ImageJ (NIH, Bethesda, MD).

### Histology and immunohistochemistry

Bladder paraffin sections (5 μm) were processed for routine hematoxylin and eosin (H&E) staining or immunohistochemistry. H&E-stained sections were evaluated by a pathologist blinded to the experimental treatment and scored for edema and inflammation according to the following scale: 0, No edema and no infiltrating cells; 1, Mild submucosal edema and no inflammatory cells; 2, Moderate edema and several inflammatory cells; 3, Frank edema, vascular congestion and many inflammatory cells.

For immunohistochemistry, batch-stained paraffin sections (N = 6/group) were blocked (5% goat serum, 0.2% Triton X-100 in PBS, 30 min at room temp.), then incubated overnight at 4°C with either rabbit polyclonal anti-HMGB1 antibody (1:100; ab18256; Abcam, Cambridge, MA) or monoclonal anti-cleaved caspase-3 (as a marker for apoptosis; 1:400 Asp175, clone 5A1E #9664; Cell Signaling Technology, Danvers, MA). Immunoreactivity was detected with goat anti-rabbit TRITC-labeled secondary antibody (1:100 in PBS with 1% goat serum, 0.2% Triton X-100; 1 hour at room temp.; Jackson ImmunoResearch, Inc., West Grove, PA) before cover-slipping (Vectashield, Vector Laboratories, Burlingame, CA).

Computer-assisted densitometry of HMGB1 immunostaining intensity was performed on images captured using a LEICA DMI4000B microscope equipped with the LAS V4 program and ImageJ (NIH, Bethesda, MD). For each bladder, intensity values represent an average of mean grey values recorded from region-of-interests (ROIs) drawn around the urothelium in three fields of view in the same section for comparisons between groups. Cleaved caspase-3 immunostained nuclei were counted from 3 random fields/animal.

### Statistical analyses

HMGB1 levels from human urothelial cell (UROtsa) culture media and intravesical fluid collected from mouse bladders exposed to PAR-AP were analyzed using planned comparisons (Student’s t-tests). Bonferroni-corrected one-tailed paired t-tests comparing positive response frequency (%) at baseline to that at 24 hours after treatment evaluated significant changes in abdominal hypersensitivity. Differences in immunofluorescence intensity were assessed in planned comparisons (Student’s t-tests).

All data are presented as mean ± SE, with statistical differences of *p* ≤ 0.05 considered significant. All statistical analyses were performed using SPSS Statistics (IBM, New York, NY).

## Results

### PAR4 activation induced HMGB1 release

**Fig 2:**
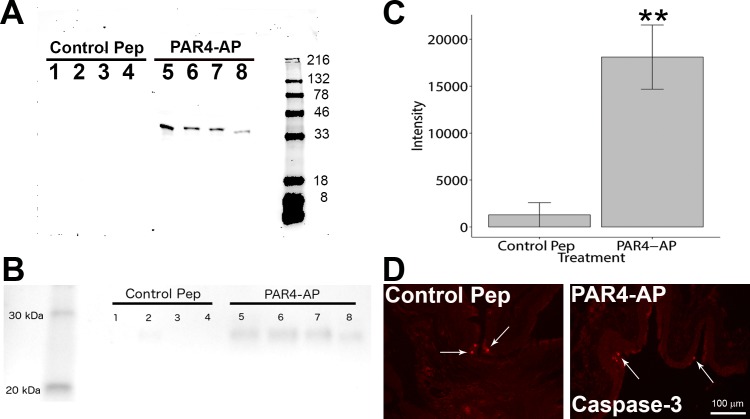
PAR4 activation elicits HMGB1 release *in vitro* and *in vivo*. Western blotting determined HMGB1 levels in culture media collected from human immortalized urothelial cultures (UROtsa) exposed to PAR4-AP for 2 hours (A; representative blot), and in intravesical fluid collected from mice receiving intravesical PAR4-AP for 1 hour (B). Densitometry revealed significantly more HMGB1 release from bladders receiving intravesical PAR4-AP than those receiving control peptide (C; ** *p* = 0.011; Wilcoxon rank test). Caspase-3 immunostaining (arrows) revealed few labeled nuclei in each treatment group (D).

Activation of urothelial PAR4 receptor elicited HMGB1 release *in vitro* and *in vivo*. HMGB1 western blotting of media collected from human immortalized urothelial cultures (UROtsa) 2 hours after receiving PAR4-AP revealed a single clear band whereas no band was detected in the media of cultures receiving control peptide (Fig 2A). The specificity of HMGB1 could be verified by the presence of a single band by HMGB1 primary antibody in the blot and confirmed by an absorption control experiment where pre-incubating the antibody with blocking peptide (ab18650; Abcam, Cambridge, MA) obtained resulted in no bands detected (data not shown). The protein loading was equal in all lanes and these were the same samples used for the above Western blot to be served as loading controls. Commassie staining of the gel (S1 Fig) and protein staining (Novex® Reversible Protein Stain; Thermo Sci., Rockford, IL) of the blot (S2 Fig) confirmed that equal amounts of protein were loaded in all lanes.

Similarly in mice, HMGB1 western blotting of intravesical fluid (using the same antibody) collected 1 hour after bladder PAR4-AP instillation revealed a single band (at the expected molecular weight 28 kDa) in every sample whereas only a very faint band was visible in samples from mice receiving intravesical control peptide (Fig 2B and 2C). Immunostaining of cleaved caspase-3, an apoptosis marker, revealed minimal expression in umbrella cells of either group, and no difference was found between treatments (Fig 2D; Control peptide, mean = 3.0 ± 2.0; PAR4-AP, mean = 3.9 ± 0.9 nuclei/mouse).

#### HMGB1 antagonist prevented PAR4-induced bladder hypersensitivity

**Fig 3:**
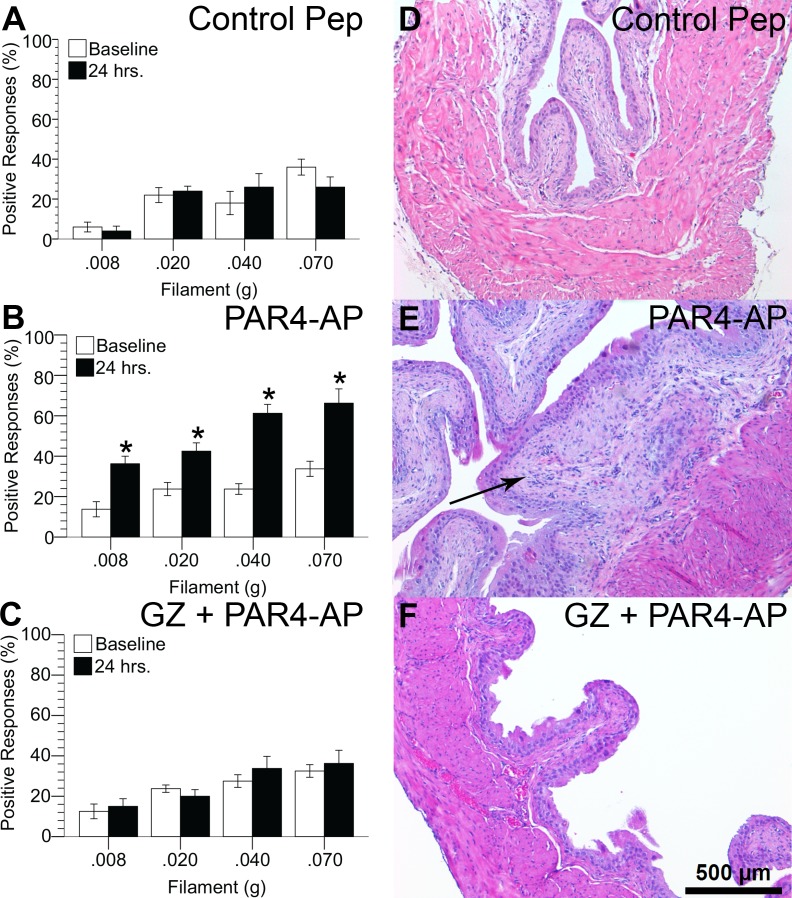
HMGB1 mediates urothelial PAR4-induced mechanical hypersensitivity without inflammation. Responses to abdominal mechanical stimulation with von Frey filaments before (baseline) and 24 hours after each treatment are shown (A-C). Intravesical PAR4-AP (B) increases abdominal hypersensitivity over corresponding baseline values (* *p* ≤ 0.025 after Bonferroni correction for multiple one-tailed paired t-tests). HMGB1 inhibitor, glycyrrhizin (50 mg/kg, ip), abolished PAR4-mediated responses (C). H&E stained paraffin bladder sections showed normal urothelial morphology in all treatment groups (D-F). No inflammatory cells were observed in any of the treatment groups. Submucosal fibrosis with lamina propria expansion (arrow in panel E) was observed in animals treated with PAR4-AP and vehicle, but not in the other groups.

We measured positive responses to von Frey filaments applied to the abdominal / perineal area before and 24 hours after instilling solutions into the bladder containing a control peptide or PAR-4AP. After 24 hours, frequency of positive responses remained the same for control peptide (Fig 3A) but increased significantly for PAR4-AP treatment (Fig 3B). PAR4-AP increased positive responses to the firmest filament (0.070 g) from 33.8 ± 3.8% before treatment (baseline) to 66.3 ± 7.1% 24 hours after treatment (*p* ≤ 0.05), indicating PAR4-AP elevated abdominal mechanical sensitivity. Pretreatment with the HMGB1 antagonist, glycyrrhizin, completely prevented the PAR4-induced hypersensitivity (Fig 3C).

We evaluated urothelial morphology, bladder edema and inflammatory infiltrates 24 hours after intravesical administration of PAR4-AP. Urothelial morphology was normal in all three groups and there was no evidence of inflammatory infiltrates in any of the treatment groups (Fig 3D–3F). Subtle stromal reactive changes (submucosal fibrosis with lamina propria expansion; Fig 3E; mean score = 0.75, SEM = 0.11) were noted only in six of eight mice treated with vehicle and PAR4-AP (mean overall score = 0.75, SEM = 0.1). These changes were not present in the groups receiving control peptide or the group receiving glycyrrhizin pre-treatment and PAR4-AP treatment (mean overall score = 0, both groups).

### MIF antagonist prevented PAR4-induced urothelial HMGB1 intensity decrease

**Fig 4:**
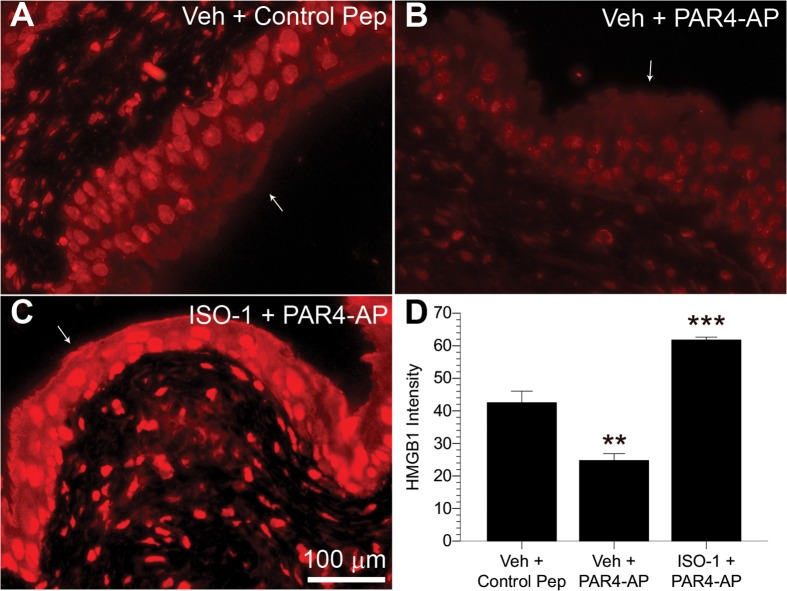
Urothelial PAR4-induced HMGB1 intensity decrease is mediated through MIF. Panels (A-C) show urothelial HMGB1 immunofluorescence 1 hour after intravesical exposure to control peptide, PAR4-AP, or PAR4-AP after pretreatment with MIF antagonist, ISO-1 (ip). White arrows identify the intravesical surface of the urothelium. Less urothelial HMGB1 immunofluorescent labeling is apparent in PAR4-AP exposed bladders (B) than in control peptide-treated bladders (A). Pretreatment with MIF-1 antagonist ISO-1 prevented this decrease (C). Quantitative image analysis (D) revealed that average urothelial HMGB1 immunofluorescence significantly decreased after intravesical PAR4-AP administration in comparison to control peptide-treated animals (** *p* ≤ 0.01), indicating urothelial release of HMGB1. In contrast, ISO-1 pretreatment elevated urothelial HMGB1 immunofluorescence from PAR4-AP administration (*** *p* ≤ 0.001), suggesting MIF antagonism blocks HMGB1 release.

Since we previously reported that MIF mediates PAR4-induced bladder hypersensitivity [7], we investigated whether MIF mediates bladder pain by modulating HMGB1 release. Strong HMGB1 immunofluorescence was detected in urothelial nuclei of mice treated with control peptide (Fig 4A), whereas intravesical PAR4-AP administration reduced urothelial HMGB1 immunofluorescence (Fig 4B) after 1 hour of exposure. Pretreatment with MIF antagonist, ISO-1, prior to intravesical PAR4 instillation, completely prevented this reduction of urothelial HMGB1 immunofluorescence (Fig 4C). Control slides where primary antisera were omitted had no immunofluorescence (not shown). Quantitative image analysis of the urothelia (Fig 4D) showed that PAR4-AP administration reduced urothelial HMGB1 by 41.7% compared to controls (*p* ≤ 0.01), whereas ISO-1 pretreatment blocked the reduction induced by bladder PAR4-AP infusion and actually elevated HGMB1 levels (Fig 4C and 4D; *p* ≤ 0.001).

## Discussion

Our results demonstrate a novel finding: activation of urothelial PAR4 receptors elicits HGMB1 release from both human and mouse urothelial cells. This conclusion is supported by the increased levels of HMGB1 seen in the intravesical fluid of mice and in the culture media of human urothelial cells following exposure to PAR4-AP. We also documented a decrease in nuclear HMGB1 in the urothelium of mice after exposure to PAR4-AP which complements the intravesical findings.

The present study, not only confirms our earlier findings [7] that PAR4 activation induced bladder pain but also extends them by showing that such bladder pain can be prevented by administration of a HMGB1 antagonist (glycyrrhizin). Our findings are in agreement with the observations of Tanaka et al. [6] who reported that HGMB1 mediated pain caused by a chemical model (cyclophosphamide) of cystitis in mice.

Lastly, since we showed that PAR4 activation induced MIF release and bladder pain that were blocked by a MIF antagonist (ISO1) [7], we tested whether MIF was upstream of HMGB1 release in our model. In fact, our current results show that a MIF antagonist (ISO1) also prevents PAR4-induced urothelial HMGB1 release. Thus, the current study provides a mechanism for our findings that PAR4 activation induces bladder pain mediated by MIF [7] by providing a mechanism (release of urothelial HMGB1) for this pain.

Our current findings using a model that results in no urothelial damage or frank inflammation provide a mechanism for MIF-mediated pain that we reported using other models of bladder inflammation (cyclophosphamide cystitis) [23]. Thus, our current and past experimental observations [7,23] indicate that MIF likely plays a pivotal role in mediating bladder pain, at least in experimental models of bladder pain and cystitis.

Therefore, we propose that activation of urothelial PAR4 by proteases (either in the urine or produced by local inflammatory events) is likely to elicit urothelial MIF release. Released MIF then binds to urothelial MIF receptors [7] resulting in HMGB1 release from urothelial cells. In turn, released HMGB1 interacts with receptors localized at nerve endings or on the urothelium relaying signals to the nerve endings to elicit bladder pain [6,24,25].

HMGB1, is a ubiquitous nuclear non-histone DNA-binding protein that signals tissue damage when passively released from cells during apoptosis [26]. Cleaved caspase-3 staining showed a few cells stained in top layer of urothelium as a regular program death of umbrella cells in all groups. Since no immunohistochemical difference (cleaved caspase-3 staining) was observed in the urothelium of PAR4-AP treated mice, we consider it unlikely that PAR4-AP treatment is causing apoptotic changes in the urothelium that account for HMGB1 release. Endogenous HMGB1 can also be actively released from cells as a result of inflammatory stimuli (e.g. LPS, TNF) to mediate further inflammation and also pain (for a review see Kato & Svensson, 2015 [8]).

The physiological activity of HMGB1 depends on the redox-state of its 3 cysteine groups [27]. In the fully reduced state (all-thiol), HMGB1 is a chemoattractant acting through the receptor for advanced glycation endproducts (RAGE). In the partially reduced state (disulfide), HGMB1 induces cytokine expression and mediates inflammation through binding with TLR4 receptor. Fully oxidized HGMB1 has no known physiological activity.

Recent evidence shows that HGMB1 can mediate pain by acting at the organ level (as is the case in this study) [6] and also at spinal levels [28,29], but the mechanisms for inducing pain are still being investigated. All-thiol HGMB1 mediates dorsal root ganglia neuronal excitability *in vitro* through RAGE receptors while the disulfide form mediates nociception at the spinal cord level *in vivo* [24]. Tanaka reported that bladder pain from cyclophosphamide injection was prevented by systemic administration of a RAGE antagonist [6] but the HMGB1 redox form was not investigated. Therefore, the redox form of HGMB1 that mediates bladder pain in our model (urothelial PAR4 activation) and the receptor for HGMB1-mediated bladder pain, remain to be investigated.

Urothelial basal and intermediate cells co-express MIF, a cytokine involved in pain and inflammatory processes [19], along with PAR1 and PAR4 receptors [16,30]. We previously showed that, when stimulated, PAR1 and PAR4 receptors elicit urothelial MIF release to mediate additional inflammatory changes and bladder pain [7,19,23,30]. The exact mechanism whereby MIF is acting as a nociceptive molecule is not known, but it is likely to involve MIF binding to one of its receptors (CD74, CXCR2 or C-X-C chemokine receptor type 4 (CXCR4) [31]). Interestingly, we previously showed that an antagonist of CXCR4 reduced bladder pain after PAR [7]. Whether, CXCR4 antagonism or antagonism of any of the other MIF receptors can prevent PAR4 induced HMGB1 release is not known but will be investigated.

In summary, our study shows that bladder pain may be modulated by disrupting several distinct molecules. Preventing urothelial PAR4 from becoming activated represents the highest point in the activation cascade sequence. Preventing the released MIF from binding one (or several) of MIF’s receptors after PAR4 activation may prevent HMGB1 release. Finally blocking released HMGB1 from binding to its receptors may block pain. The contribution of intracellular signaling pathways is unclear and needs to be studied further. These events offer multiple control points that may offer therapeutic targets to relieve bladder pain in clinical conditions such as IC/PBS. Whether these molecules are also elevated in clinical conditions remains to be determined. IC/PBS patients show elevated urine protease levels [17,18], which presumably may lead to greater bladder PAR activation, and in turn, bladder hypersensitivity. Our model of intravesical administration of PAR peptides to induce bladder pain may be replicating this process [7].

## Conclusions

Intravesical stimulation of bladder PAR4 receptors induced bladder pain in this study by eliciting urothelial HMGB1 release through a MIF-mediated mechanism. These findings suggest that MIF, acting upstream of HMGB1, plays a key role in mediating bladder pain. Both molecules represent novel targets for therapeutic intervention in bladder pain conditions. Future studies will examine the contribution of specific receptors activated by MIF and HMGB1 in mediating bladder pain.

## Supporting Information

S1 Fig Commassie-stained gel shows equivalent protein in each lane. Human epithelial cells (UROtsa) culture media (from samples used in Fig 2A) were loaded on a gel, electrophoresed and stained for protein using a Commassie procedure.
(JPG)

S2 Fig Protein-stained gel shows equivalent protein in each lane. Human epithelial cells (UROtsa) culture media (from samples used in Fig 2A) were loaded on a gel, eletrophoresed and stained for protein.
(JPG)

S1 File Raw data used for the analyses and figures. (ZIP)

## Abbreviations

CXCR4: C-X-C chemokine receptor type 4
GZ: glycyrrhizin
H&E: hematoxylin and eosin
HMGB1: high-mobility group box 1 protein
IC/PBS: Interstitial Cystitis / Painful Bladder Syndrome
ISO-1: (S,R)3-(4-hy-droxyphenyl)-4,5-dihydro-5-isoxazole acetic acid methyl ester
MIF: macrophage migration inhibitory factor
PAR: protease activated receptor
PAR4: protease activated receptor 4
PAR4-AP: PAR4-activating peptide
Pep: peptide
RAGE: receptor for advanced glycation endproducts
ROI: region of interest
TLR4: toll-like receptor 4
Veh: vehicle
