# Supplementary figures and images for: Protease-Activated Receptor 4 Induces Bladder Pain through High Mobility Group Box-1
Source: PLoS One. 2016 Mar 24;11(3):e0152055. doi: 10.1371/journal.pone.0152055 (PMC4806866; doi:10.1371/journal.pone.0152055)

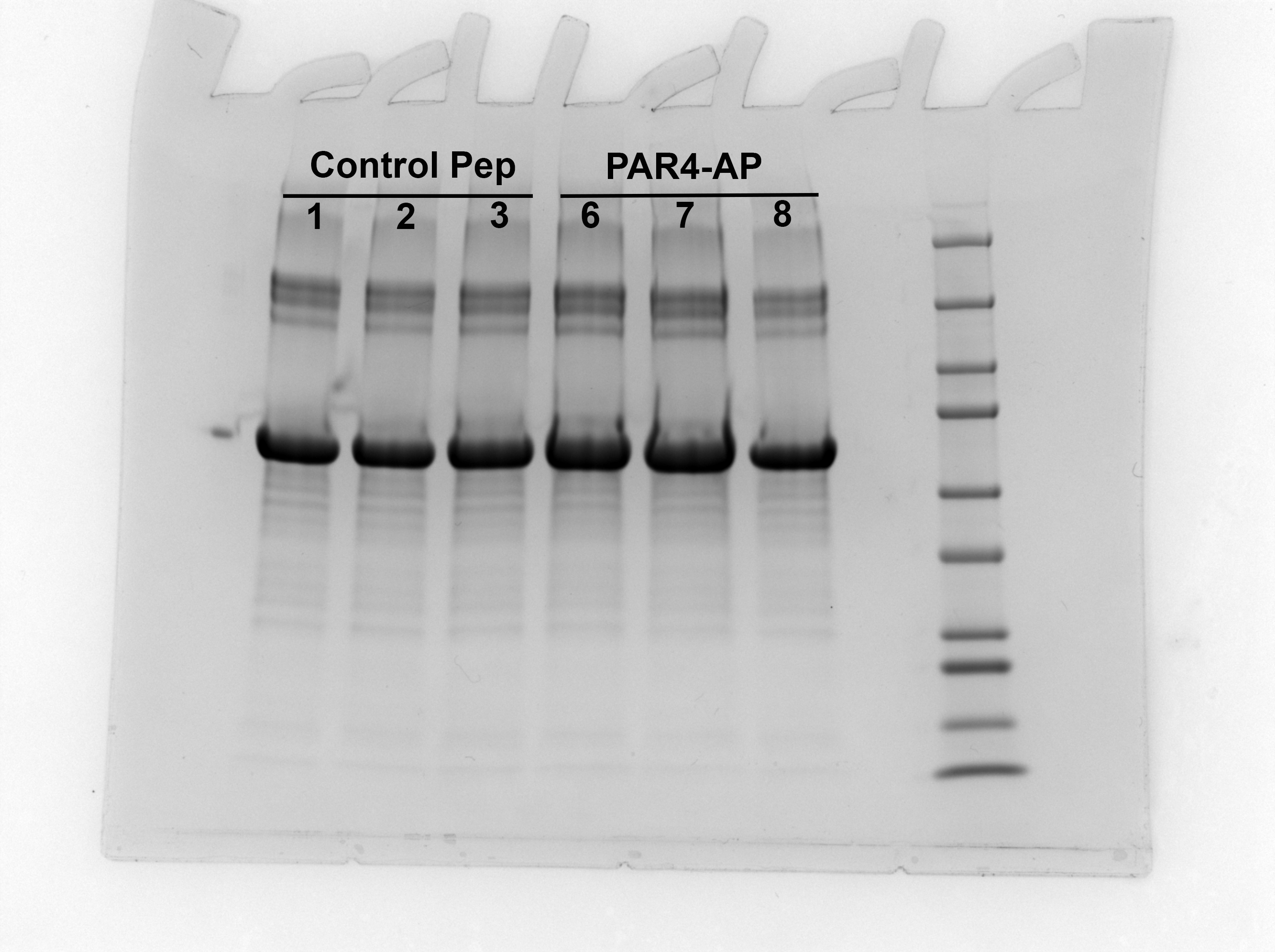

Supplement: S1 Fig — Human epithelial cells (UROtsa) culture media (from samples used in Fig 2A) were loaded on a gel, electrophoresed and stained for protein using a Commassie procedure. (JPG) [file pone.0152055.s001.jpg]

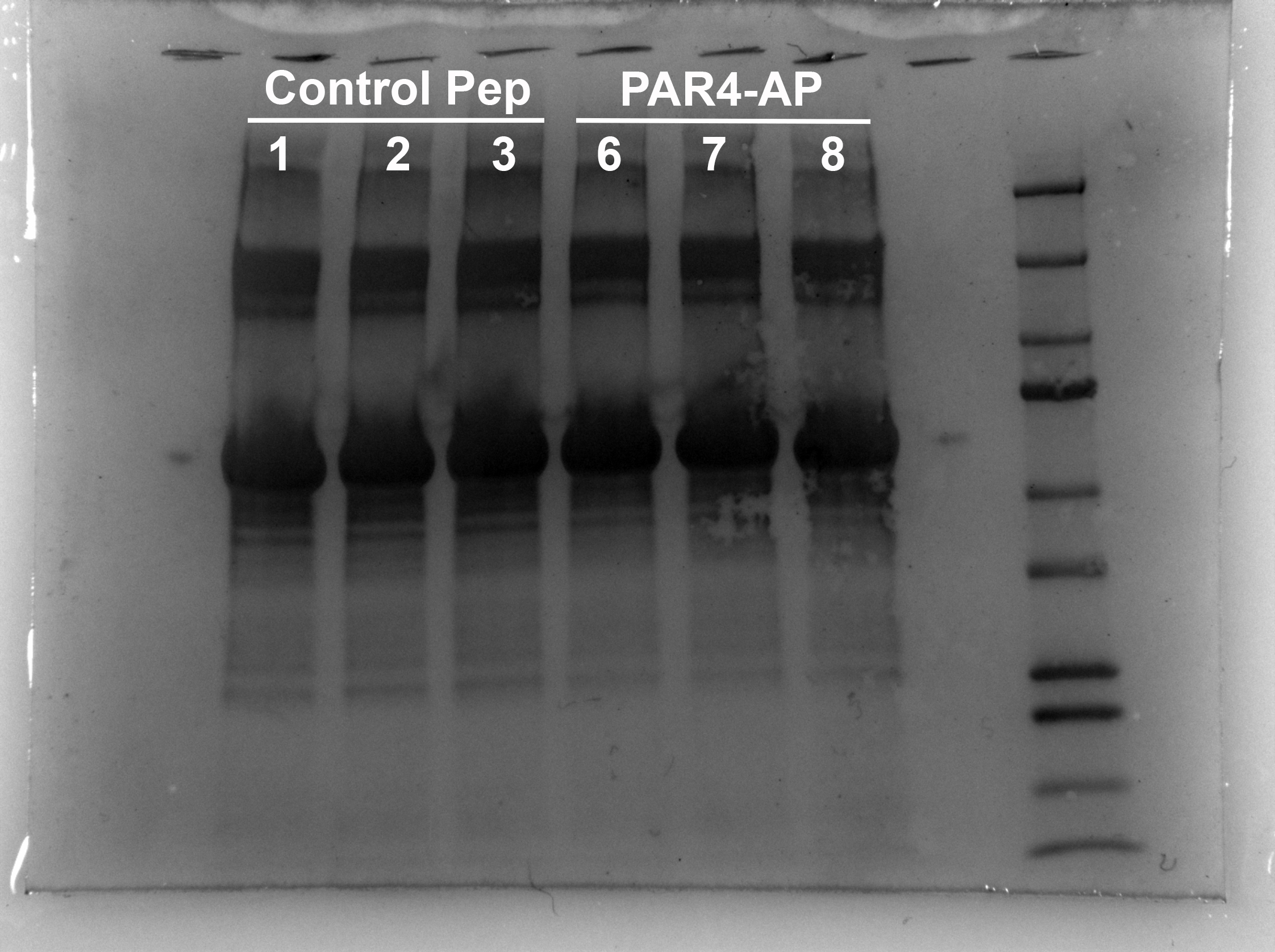

Supplement: S2 Fig — Human epithelial cells (UROtsa) culture media (from samples used in Fig 2A) were loaded on a gel, eletrophoresed and stained for protein. (JPG) [file pone.0152055.s002.jpg]
